# Agreement and Reliability Between Tele-Assessment and In-Person Assessment of the One-Minute Sit-to-Stand Test in Patients with Chronic Respiratory Diseases

**DOI:** 10.3390/jcm14145049

**Published:** 2025-07-16

**Authors:** Santiago Larrateguy, Matías Otto-Yáñez, Juan Bogado, Luis Larrateguy, Marisol Barros-Poblete, Guillermo Mazzucco, Isabel Blanco, Elena Gimeno-Santos, Rodrigo Torres-Castro

**Affiliations:** 1 Facultad de Ciencias de la Salud, Universidad Adventista del Plata, Libertador San Martin 3103, Argentina; santilarra@gmail.com; 2 Centro Privado de Medicina Respiratoria, Paraná 3100, Argentina; juannbtrabajo@gmail.com (J.B.); ldlarrateguy@gmail.com (L.L.); 3 Grupo de Investigación en Salud, Funcionalidad y Actividad Física (GISFAF), Kinesiología, Facultad de Ciencias de la Salud, Universidad Autónoma de Chile, Santiago 8370024, Chile; matiasotto.kine@gmail.com; 4 Universidad Austral de Chile, Valdivia 5090000, Chile; mjbarrosp@gmail.com; 5 Deusto Physical TherapIker, Physical Therapy Department, Faculty of Health Sciences, University of Deusto, 20012 Donostia-San Sebastián, Spain; guillermo.mazzucco@deusto.es; 6 Unidad de Rehabilitación Cardiopulmonar, Ammma, 20014 Donostia-San Sebastián, Spain; 7 Department of Pulmonary Medicine, Hospital Clínic–Institut d’Investigacions Biomèdiques August Pi i Sunyer (IDIBAPS), University of Barcelona, 08036 Barcelona, Spain; iblanco2@clinic.cat; 8 Barcelona Institute for Global Health (ISGlobal), 08003 Barcelona, Spain; elenagimeno@gmail.com; 9 Department of Physical Therapy, Faculty of Medicine, University of Chile, Santiago 8380453, Chile

**Keywords:** physical capacity, field tests, tele-assessment, sit-to-stand test, pulmonary rehabilitation

## Abstract

**Background/Objectives:** Telemedicine has emerged as a valuable tool for overcoming access barriers in healthcare, particularly in rehabilitation. However, the validity and reliability of remotely conducted physical capacity assessments remain unclear. This study evaluated the agreement and intra-rater reliability between in-person and tele-assessment administration of the one-minute sit-to-stand test (1 min-STST) in individuals with chronic respiratory diseases (CRDs). **Methods:** In this cross-sectional study, forty adults (55% female; mean age 59.8 ± 15.9 years) diagnosed with CRDs—including chronic obstructive pulmonary disease (52.5%), asthma (20%), and pulmonary fibrosis (20%)—completed the 1 min-STST in two conditions: in person and via tele-assessment. The primary outcome was the number of repetitions completed in each condition. Intra-rater reliability was analyzed using the intraclass correlation coefficient (ICC), and agreement between methods was evaluated with Bland–Altman analysis. **Results:** The mean number of repetitions was 24.4 ± 8.0 in person and 24.3 ± 8.1 via tele-assessment, with no significant difference (*p* = 0.78). Excellent reliability was observed (ICC = 0.978, *p* < 0.001), and Bland–Altman analysis showed good agreement with a mean difference of 0.08 ± 1.7 repetitions and limits of agreement from −3.26 to 3.41. No adverse events were reported. **Conclusions:** Tele-assessment of the 1 min-STST shows excellent agreement and reliability compared to in-person assessment in individuals with CRDs. These findings support tele-assessment as a valid and practical alternative for evaluating functional capacity remotely. Further research is needed to confirm its implementation in home-based or less-controlled settings.

## 1. Introduction

Given the characteristics of the COVID-19 pandemic and the need for social distancing, incorporating telemedicine resources in rehabilitation became a logical solution to deliver healthcare to the population [1]. Since then, it has become an essential tool in various specialties, leading to the development of related concepts such as telerehabilitation [2].

Although studies have indicated that the implementation of telerehabilitation programs is comparable with conventional in-person rehabilitation in positive clinical results [3], it is necessary to establish which interventions are helpful, safe, and valid in this context, specifically those evaluations that can be developed remotely [4].

In this context, functional capacity, particularly physical capacity assessment has been widely reported to be useful in rehabilitation programs in determining the effectiveness of programs, prescribing exercise, and setting entry priority [5]. The gold standard for assessing physical capacity is the cardiopulmonary exercise test (CPET), as it provides comprehensive physiological data [6]. However, its implementation is limited by high costs, the need for specialized equipment, and trained personnel [6]. As a result, field tests such as the 6 min walk test (6MWT) have gained popularity. These tests offer a practical alternative for evaluating functional capacity in low-resource settings [7].

The 6MWT has been commonly used as a standard to evaluate functional capacity in different population groups due to its simplicity and effectiveness [7]. However, in scenarios where the 6MWT cannot be performed, such as in the telerehabilitation context, the one-minute sit-to-stand test (1 min-STST) is a viable alternative [8].

In other pathologies, primarily respiratory, the 1 min-STST has demonstrated a high correlation with 6MWT with good psychometric properties [8,9,10]. It is even recommended as an alternative to the 6MWT and has shown better performance than other tests indeed, such as the Chester Step Test [11,12,13], although they were different tests and required different movements. Morita et al. evaluated oxygen consumption and clinical correlations across different variations in the sit-to-stand test protocols in individuals with COPD. Among the 5-repetition, 30 s, and 1 min versions, the 1 min-STST demonstrated the strongest correlation with the 6MWT (r = 0.40) and with daily physical activity metrics. It also elicited greater physiological responses—including changes in oxygen saturation, heart rate, and perceived exertion—making it more comparable in demand to the 6MWT. These findings suggest that the 1 min-STST is the most suitable STS protocol for estimating functional capacity in settings where full cardiopulmonary testing is not feasible [14].

The 1 min-STST is particularly valuable in evaluating and monitoring people with respiratory, metabolic, or cardiovascular conditions, offering valuable information about their functional capacity and the effectiveness of treatments and therapies applied [11]. As a result, the 1 min-STST has become increasingly popular in rehabilitation settings due to its practicality and ease of use, especially in primary care and telerehabilitation contexts. It allows for the assessment and follow-up of individuals with physical impairments in a more accessible manner [15]. These approaches make it possible to perform field-based evaluations like the 1 min-STST remotely, supporting ongoing patient monitoring and progress evaluation [12]. This not only enhances the rehabilitation process but also contributes to improved quality of care for both patients and healthcare providers [12,16].

Tele-assessment methods such as the remote administration of the 1 min-STST have been identified as safe, acceptable, and feasible in patients with chronic respiratory diseases. These approaches enable continued monitoring and foster patient engagement and empowerment in the self-management of long-term conditions like COPD, where access to traditional services is often limited [17].

Furthermore, integrating the 1 min-STST into telemedicine strategies may support tailored rehabilitation plans. Its performance has been linked to better physical function and health-related quality of life [18], highlighting its utility in prioritizing individualized interventions. In a broader context, the STST has been suggested as a functional screening tool to guide preventive and rehabilitative efforts under personalized medicine frameworks [19].

Ultimately, the evaluation of remote STST reliability contributes to advancing tertiary prevention strategies, facilitating early detection of functional decline and developing customized care pathways in individuals with chronic diseases like COPD [20].

Despite the growing use of the 1 min-STST in remote rehabilitation, there is still limited evidence evaluating its measurement properties when administered through tele-assessment, particularly in comparison to face-to-face administration. Most studies have focused on feasibility and patient perceptions, with fewer assessing psychometric aspects such as inter- and intra-rater reliability or agreement between modalities. This gap highlights the need for studies validating the equivalence of remote versus in-person testing procedures, which is essential to ensure clinical confidence, safety, and consistency in data interpretation across settings. This study aimed to evaluate the agreement and reliability between the in-person evaluation and the tele-assessment of the 1 min-STST in chronic respiratory diseases (CRDs).

## 2. Methods

### 2.1. Study Design and Participants

A cross-sectional study was conducted in patients with CRDs. Recruitment was conducted within a respiratory rehabilitation unit at a Private Center for Respiratory Medicine in Paraná, Argentina, from February 2023 to April 2023. This study was approved by the Ethics Committee (Independent Research Ethics Committee, code: CE000344, 17 February 2023). All subjects gave written consent. This study conforms to all STROBE (Strengthening the Reporting of Observational Studies in Epidemiology) guidelines and reports the required information accordingly [21].

Patients registered in the private respiratory medicine center were invited to participate in this research. The inclusion criteria were adults between 20 and 80 years of age, diagnosis of CRDs (Chronic Obstructive Pulmonary Disease (COPD), asthma, obstructive sleep apnea (OSA), idiopathic pulmonary fibrosis, e.g.) who reported being capable of rising from and sitting back down on a chair, have pulmonary function study records no more than 6 months old and agree to participate and sign the informed consent. The exclusion criteria were: a body mass index (BMI) ≥ 35, a respiratory exacerbation in the last 30 days, an acute or chronic musculoskeletal injury, or the presence of concomitant cardiac, cerebral, or neuromuscular conditions that hinder test performance, and/or presenting inability to understand instructions.

Participants were instructed not to engage in vigorous physical activity and to avoid caffeine intake within 2 h prior to testing. Prior to enrollment, patients were screened for internet access and the ability to use video conferencing software, ensuring feasibility of remote participation. Sociodemographic data were also collected to evaluate potential factors influencing test performance or feasibility of tele-assessment.

### 2.2. Measurements

**Figure 1:**
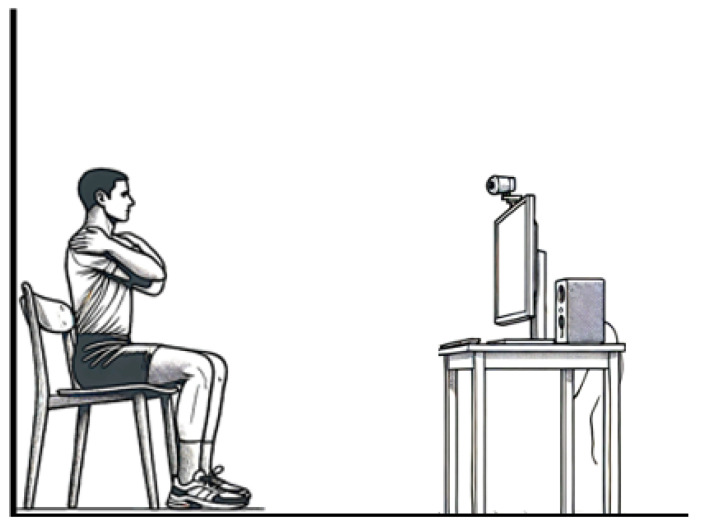
Schematic representation of the 1 min-STST tele-assessment procedure.

Each participant completed all assessments during a single visit following a predetermined standardized sequence. During the visit, demographic and anthropometric characteristics were collected. Additionally, the most recent spirometry conducted at the medical center within the previous six months was included in the data collection. Spirometry was performed by a trained technician following the American Thoracic Society/European Respiratory Society (ATS/ERS) guidelines to ensure standardization and accuracy of the measurements [22]. The in-person and tele-assessment via video call took place in separate rooms. These rooms varied in their setups: the room designated for video call evaluation was furnished with a desk, a computer featuring a video camera and speakers, designed to facilitate the inconspicuous conduction of the non-supervised test through the video software (Zoom Video Communications, version 5.13.10, San José, CA, USA) (Figure 1). The sequence of evaluations was determined through a process of simple randomization utilizing open-source software. A standardized rest of 30 min between the tests was included [11]. Both assessments were conducted by the same evaluator in a randomized order.

To minimize variability in remote conditions, participants were provided with detailed written and verbal instructions in advance, including recommendations on chair type and positioning, device placement for self-monitoring, and safety precautions during testing. Additionally, prior to the tele-assessment, a brief training session was conducted to familiarize participants with the procedure and ensure the correct setup. A protocol checklist was completed by the evaluator in both modalities to standardize implementation and facilitate protocol adherence monitoring.

The use of the same evaluator for both test modalities aimed to reduce inter-rater bias. The 30 min rest period was monitored to ensure a return to baseline hemodynamic parameters before the second test.

The 1 min-STST requires the patient to transition from a seated position to a fully upright standing position, adopting the bipedal position with their knees fully extended [23,24]. A standard chair with a seat height of 46 cm with thoracolumbar back support was used [25,26]. Subjects were seated upright on the chair against the wall with knees and hips flexed at 90° and feet resting on the floor wide apart [27]. The primary outcome was the number of complete sit-to-stand repetitions performed within one minute, defined by fully seated and fully upright positions. Only a single 1 min-STS test was administered [28]. Before and after the 1 min-STST, subjects were asked about dyspnea and lower limb fatigue according to the modified Borg scale [29]. Peripheral oxygen saturation (SpO_2_), heart rate (HR), and heart rate recovery at one minute (HRR) were measured and registered using an oximeter device (Nonin Inc., Plymouth, MN, USA). While the Borg scale was administered at remote assessment, SpO_2_ and HR measurements were undertaken by the patient’s self-monitoring. The same oximeter device was utilized for both evaluations to minimize inconsistencies related to measurement instruments.

### 2.3. Statistical Analysis

Statistical analyses were performed using IBM SPSS Statistics for Mac (Version 25.0, IBM Corp: Armonk, NY, USA) and JASP v0.18.3 (JASP Team, Amsterdam, The Netherlands). The normal distribution of the data was assessed using the Shapiro–Wilk test. Descriptive statistics were employed, including mean and standard deviation, median and interquartile range for quantitative variables. For qualitative variables, frequencies and percentages were utilized. Paired *t*-tests or Wilcoxon signed-rank tests were used to compare continuous variables between in-person and tele-assessment conditions, depending on the distribution of the data. Effect sizes (Cohen’s d) and their 95% confidence intervals were calculated for each outcome using paired-sample statistics. The effect size was categorized as small, moderate, and large, with Cohen’s d values < 0.2, between 0.2 and 0.5, and >0.8, respectively [30].

To ascertain the level of agreement between the in-person and tele-assessment, the intra-rater reliability was determined by calculating the intraclass correlation coefficient (ICC). The ICC was calculated using a two-way mixed-effects model for single measures and absolute agreement [ICC(3,1)], as recommended for intra-rater comparisons involving fixed raters. The ICC was interpreted according to standard reliability thresholds: low (<0.5), moderate (between 0.5 and 0.75), good (between 0.75 and 0.90), and excellent (>0.90) [31]. Additionally, Bland–Altman analysis was conducted to assess the level of agreement between the in-person 1 min-STST and the tele-assessment 1 min-STST.

The standard error of measurement (SEM) was computed using the formula: SEM = SD × √(1 − ICC), where SD corresponds to the standard deviation. To estimate the minimal detectable change at a 95% confidence level (MDC95), the following equation was applied: MDC95 = SEM × 1.96 × √2. The SEM represents the degree of error inherent in repeated measurements, while the MDC defines the minimal amount of change that must be observed to be considered real and not due to measurement variability. These indices serve as reference values to assess whether variations in scores are both statistically significant and clinically relevant [32,33]. To reinforce the robustness of the comparison between in-person and tele-assessment conditions, a bootstrap analysis with 1000 resamples was conducted to estimate the confidence interval of the mean difference in 1 min-STST performance, providing a non-parametric inference of equivalence.

Although a sample size of 30 participants was sufficient to achieve an expected ICC of 0.85 with a 95% confidence interval width no greater than 0.199 [34], this calculation was based on the method proposed by Giraudeau & Mary [22], which estimates the required number of subjects by targeting a desired level of precision for the ICC confidence interval. This approach, designed explicitly for reproducibility studies with a single sample, allows determining the sample size without relying on hypothesis testing but instead on the expected width of the 95% CI. To improve the estimate’s precision and enhance the sample’s stability and representativeness, we included 40 participants in this study, exceeding the minimum requirement and accounting for potential technical errors or missing data.

## 3. Results

Data were collected from a cohort of 40 participants, of which 22 were female (55%). The mean age was 59.8 ± 15.9 years, accompanied by an average height of 1.69 ± 0.05 m, weight of 79.0 ± 18.3 kg, and a BMI of 28 ± 6.6 kg/m^2^. Regarding diagnoses, 21 individuals were assessed with COPD (52.5%), eight presented asthma (20%), eight with pulmonary fibrosis (20%), and one each with tuberculosis (2.5%), pulmonary hypertension (2.5%), and OSA (2.5%). Table 1 provides more information on the demographic and clinical profile of the participants.

The mean values of the in-person and the tele-assessment 1 min-STST were 24.4 ± 8.0 and 24.3 ± 8.1, respectively (Table 2). No statistically significant differences were found between these measurements (*p* = 0.78). The mean difference was 0.075 repetitions (95% CI: –0.469 to 0.619), with a small effect size (Cohen’s d = 0.044, SE = 0.034), indicating a negligible difference between modalities. This was further supported by bootstrap analysis (1000 resamples), which yielded a mean difference of 0.085 repetitions with a 95% confidence interval of –0.425 to 0.600, reinforcing the minimal variability between testing formats. No adverse events were observed.

The 1 min-STST by tele-assessment shows excellent intra-rater reliability compared to in-person for male (ICC = 0.976, *p* < 0.001) and female participants (ICC = 0.975, *p* < 0.001). The total sample also showed excellent reliability (ICC = 0.978, *p* < 0.001). The ICC for the main diagnoses was as follows: COPD = 0.972 (*p* < 0.001); asthma = 0.992 (*p* < 0.001), and pulmonary fibrosis = 0.830 (*p* = 0.01).

**Figure 2:**
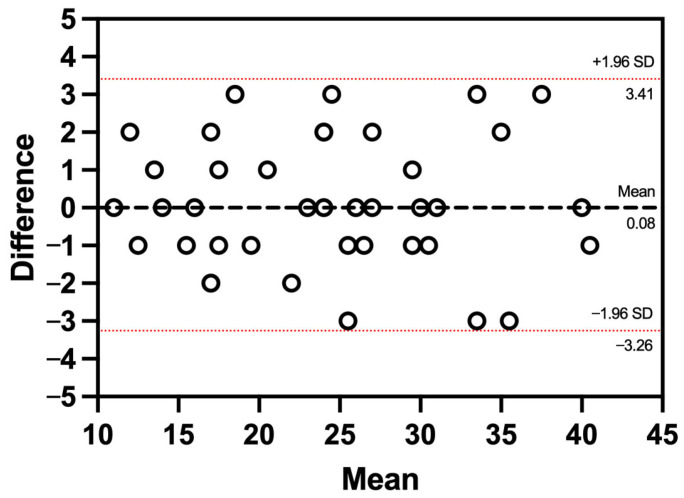
Bland–Altman Plot to assess agreement between in-person and tele-assessment evaluation. The dashed line represents the mean difference between methods (bias = 0.08 repetitions), and the red dotted lines indicate the 95% limits of agreement (−3.26 to 3.41 repetitions), calculated as mean ± 1.96 × SD of the differences.

The Bland–Altman plot showed minimal bias and acceptable agreement between remote and face-to-face assessments of the 1 min-STST, with all values falling within clinically acceptable limits (Figure 2). The average difference was calculated at 0.08 ± 1.7. The agreement ranged from 3.41 to −3.26, respectively.

The SEM for the 1 min-STST was 1.19 repetitions, and the MDC95 was 3.30 repetitions. These values represent the minimum amount of change required to be confident that an observed difference exceeds measurement error and reflects a true change in performance.

## 4. Discussion

This study supports tele-assessment as a reliable and consistent method compared to the in-person evaluation of the 1 min-STST. These findings suggest that remote assessment may be feasible for estimating physical capacity in individuals with CRDs.

The importance of telemedicine cannot be overstated. It offers a solution to distance, mobility, and resource allocation challenges, especially pertinent in CRDs, where regular monitoring and evaluation are crucial for disease management [1]. The global health crisis caused by COVID-19 triggered the widespread adoption of telemedicine, showcasing its versatility and effectiveness in delivering care remotely [1]. Therefore, integrating teleassessment in the rehabilitation field into the daily practice of care for people with CRDs represents an opportunity to improve patient-centered care while optimizing healthcare resources [12]. Telemedicine has emerged as a valuable strategy for enhancing self-management in patients with chronic respiratory diseases. A comprehensive review by Cruz et al. [35] emphasized that telehealth interventions can effectively engage patients in their own care, especially those with COPD who often face accessibility challenges to traditional healthcare services. Importantly, the review concluded that more intensive and structured telemedicine programs were associated with better clinical outcomes and higher satisfaction levels among users, underscoring the potential of virtual tools in respiratory rehabilitation programs [17].

Moreover, Crook et al. [20] demonstrated that performance on the 1 min-STST is significantly associated with important clinical outcomes in patients with COPD, including health-related quality of life, dyspnea, and physical function. Their results support the STST as a valid and responsive measure of functional status, with potential applicability in both clinical trials and routine care. Integrating the 1 min-STST into telemedicine strategies may thus offer a feasible and meaningful way to monitor patient-centered outcomes remotely, particularly in individuals with limited access to conventional rehabilitation settings [20].

Although we did not find other studies comparing intra-rater reliability between in-person and tele-assessment 1 min-STST, there are studies available for functional tests, commonly used in rehabilitation, such as the five times sit-to-stand test (5-STST) and the timed up and go (TUG) [36,37]. Ozsoy et al. reported excellent intra-rater reliability for the TUG (0.976) and the 5-STST (0.974) in patients with COPD [36]. The same group replicated the research in older adults with similar results (TUG: 0.973, 5-STST: 0.948) [37]. When considering tests of longer duration, such as the 30 s sit-to-stand test (30 s-STST), the literature has also demonstrated comparable findings, although in populations with diabetes mellitus [38].

The results have also been positive in physical capacity assessments, such as the 6MWT, although they are more difficult to implement due to the requirement of a 30 m hallway. For example, Pepera et al. reported that conducting the 6MWT outdoors via tele-assessment may be a valid and reliable method for evaluating functional capacity in individuals with type 2 diabetes [39]. Although it showed reliability, it must be considered that not all patients will have 30 m available outside their home to perform a test like this.

Although the ICC for CRDs as a whole is high, there were slight differences between them, with higher values in COPD and asthma compared to patients with pulmonary fibrosis. Our sample size and the age differences between individuals with different diagnoses do not allow us to establish potential causes. However, future studies could explore these differences.

The high values of ICC obtained in this study underscore the robust agreement between in-person and tele-assessment administration of the 1 min-STST [40]. This strong agreement reaffirms the validity and reliability of tele-assessment, reassuring both healthcare professionals and patients. Additionally, it opens up opportunities for remote monitoring and evaluation, particularly in rehabilitation, enabling timely intervention and personalized care delivery. In a systematic review and meta-analysis, Muñoz-Bermejo et al. [18] found that sit-to-stand tests demonstrate high test–retest reliability across healthy adults and various clinical populations. The study validated the STS as a robust tool for assessing lower limb strength, balance, and functional mobility—dimensions that are crucial for monitoring progression and outcomes in both in-person and tele-assessment modalities [18].

The SEM for the 1 min-STST was 1.19 repetitions, and the MDC95 was 3.30 repetitions. These values represent the minimum amount of change required to be confident that an observed difference exceeds measurement error and reflects a real change in performance. Importantly, the mean difference between in-person and tele-assessment conditions was only 0.075 repetitions—well below the MDC95 threshold—suggesting that the test yields highly consistent results across both modalities. This reinforces the clinical utility of the 1 min-STST via tele-assessment as a reliable alternative for functional evaluation in individuals with chronic respiratory diseases. Our MDC95 was slightly higher than that reported by Sevillano-Castaño et al. [41] in individuals with long COVID (MDC95 = 2.71 repetitions) but lower than the value reported by Panjiyar et al. [42] in patients with interstitial lung disease, including idiopathic pulmonary fibrosis (MDC95 = 4.60 repetitions; SEM = 1.73). These comparisons suggest that our tele-assessed 1 min-STST yields a measurement error within an acceptable and expected range across diverse clinical populations, reinforcing its reproducibility and supporting its application in remote assessment contexts.

In addition to measurement agreement, effect size analysis reinforces the negligible difference between modalities. Cohen’s d for the comparison between in-person and tele-assessment conditions was 0.044, which is considered a small effect size, indicating minimal practical difference. Effect size is particularly relevant when statistical differences are absent, as it provides a complementary understanding of the magnitude of observed effects. In this context, the trivial effect size confirms the practical equivalence of both testing approaches and strengthens the argument for the clinical utility of tele-assessment. This finding gains further significance when contrasted with Nguyen et al. [43], who reported significant learning effects between repeated administrations of the 1 min-STST in patients with heart failure, with Cohen’s d values of 1.24 and 1.78 within the same visit. In contrast, the negligible effect in our study highlights the stability and reliability of the test when applied across modalities rather than over time, supporting its reproducibility and minimizing concerns related to learning bias in remote assessment contexts.

A key implication of this study is its capacity to reduce inequities in healthcare delivery by extending services to individuals who face barriers in accessing conventional care environments. Individuals residing in remote or underserved areas that need functional assessments and those facing mobility constraints stand to benefit significantly from teleassessment initiatives [44]. By removing geographical barriers and offering convenient access to healthcare services, telemedicine can democratize healthcare, particularly rehabilitation interventions, and improve health outcomes across diverse populations [44].

Although the agreement and reliability showed good results, clinical applicability is key. Clear instructions are essential to ensure both proper implementation and safety. While the aim of this study was not to establish a guideline for performing the sit-to-stand test via telemedicine, there are recommendations which allow the test to be conducted in a controlled and safe environment [45]. Undoubtedly, scientific societies must advance in this direction to ensure the replicability of these tests.

To strengthen these findings, a bootstrap analysis with 1000 iterations was performed to estimate the sampling distribution of the mean difference between modalities. The bootstrapped mean was 0.085 repetitions, with a 95% confidence interval ranging from –0.425 to 0.600 repetitions. These results support the robustness of the primary findings, confirming that the observed difference is both statistically non-significant and clinically negligible. Employing non-parametric bootstrapping enhances the credibility of the observed equivalence by reducing data distribution assumptions and increasing the results’ generalizability.

However, despite its promise, the widespread implementation of teleassessment in the rehabilitation field warrants careful consideration of several factors. Technical limitations, such as connectivity issues and device accessibility, may hinder the seamless execution of tele-assessments, particularly in resource-limited settings [46]. Moreover, ensuring the privacy and security of patient data in the digital realm remains paramount, necessitating robust regulatory frameworks and technological safeguards. Finally, although the results indicate that it is possible to perform the test remotely, patients at home may experience orthostasis, dizziness, or other conditions that increase the risk of falls. Therefore, a pretest screening for potential safety concerns should be included.

### Limitations and Strengths

Our study has limitations; the sample size is small, although deemed sufficient according to our sample size calculation. On the other hand, the test was conducted in a clinical setting where both arms followed a standardized protocol. However, the variability in patients’ home environments poses a significant challenge to standardization. Differences in chair height, firmness, presence or absence of armrests, and floor conditions may all influence the test’s outcomes. Additionally, external factors such as the presence of pets or children could further compromise the consistency of the test. Additionally, some CRDs are underrepresented. Future studies should investigate other CRDs not included here, such as non-CF bronchiectasis.

One of the main strengths of this study is its rigorous methodological design, including standardized procedures for both in-person and tele-assessment conditions, which enhances the internal validity of the findings. The inclusion of patients with different CRDs also adds to the generalizability of the results within this population. Additionally, the study addresses a relevant and timely clinical question, filling a gap in the literature regarding the reliability of the 1 min-STST in telehealth contexts. By demonstrating strong agreement and high ICC, this work provides solid evidence to support the integration of simple, low-cost, and accessible tools like the 1 min-STST into remote rehabilitation strategies. This may be particularly valuable for optimizing patient care in under-resourced settings and for individuals with limited mobility or access to in-person services.

## 5. Conclusions

This study demonstrates excellent agreement and reliability between tele-assessment and in-person assessment of the 1 min-STST. The findings support the potential use of tele-assessment to evaluate physical capacity in individuals with CRDs remotely.

While our findings support the potential integration of the 1 min-STST into telehealth strategies, especially in resource-limited or geographically remote settings, it is important to interpret these results with caution. The study was conducted under controlled conditions, and the generalizability of the results to more diverse real-world environments—particularly those with significant variability in space, equipment, or supervision—remains to be fully explored.

Further research is needed to confirm these findings in larger and more heterogeneous populations, to establish normative values for remote assessment, and to develop specific safety protocols for unsupervised settings. Nevertheless, the present results contribute to the growing body of evidence supporting the role of tele-assessment in pulmonary rehabilitation and highlight the 1 min-STST as a promising tool for extending the reach of functional evaluation in CRDs.

## Figures and Tables

**Table 1:** Characteristics of the population.

Parameters	*n* = 40
Sex (Female/Male)	22/18
Age (years)	59.8 ± 15.9
Height (m)	1.69 ± 0.05
Weight (kg)	79.0 ± 18.3
BMI (kg/m^2^)	27.8 ± 6.6
*Diagnoses*	
COPD	21 (52.5%)
Asthma	8 (20%)
Pulmonary Fibrosis	8 (20%)
OSA	1 (2.5%)
PAH	1 (2.5%)
TBC	1 (2.5%)
*Pulmonary function*	
FEV_1_ (% pred.)	59.3 ± 21.1
FVC (% pred.)	75.9 ± 17.7
FEV_1_/FVC (%)	64.8 ± 21.9

Abbreviations: BMI: Body mass index; COPD: Chronic obstructive pulmonary disease; FEV_1_: Forced expiratory volume in the first second; FVC: Forced vital capacity; OSA: Obstructive sleep apnea; PAH: Pulmonary arterial hypertension.

**Table 2:** Clinical variables observed during the comparison.

Parameters	Face-to-Face	Virtual	*p*
Repetitions	25.1 ± 7.8	25.0 ± 8.0	0.844
SpO_2_ pre (%)	96 (94–98)	97 (94–98)	0.420
SpO_2_ post (%)	94 (90–97)	95 (90–97)	0.895
HR pre (bpm)	83 (77–89)	80 (74–90)	0.253
HR post (bpm)	105 (100–118)	109 (99–120)	0.122
Final Borg dyspnea	2 (1–3)	2 (1–3)	0.796
Final Borg lower limb fatigue	1 (0–3)	1 (0–3)	0.861
1 min-HRR (bpm)	16.2 ± 9.3	18 ± 9.0	0.074

Values are presented as mean ± standard deviation (SD) or median and interquartile range (q1–q3), as appropriate. Paired *t*-tests were used for normally distributed continuous variables, and Wilcoxon signed-rank tests for non-normally distributed data. Abbreviations—HR: Heart rate; HRR: Heart rate recovery; SpO_2_; Oxygen saturation. Data are expressed in the interquartile range (q1–q3) or mean ± SD.

## Data Availability

The datasets generated during and/or analyzed during the current study are available from the corresponding author on reasonable request.
